# Gain Control in Predictive Smooth Pursuit Eye Movements: Evidence for an Acceleration-Based Predictive Mechanism

**DOI:** 10.1523/ENEURO.0343-16.2017

**Published:** 2017-05-26

**Authors:** Lukas Brostek, Thomas Eggert, Stefan Glasauer

**Affiliations:** 1 Center for Sensorimotor Research, Ludwig-Maximilians-Universität Münich, 81377 Münich, Germany; 2 Department of Neurology, Ludwig-Maximilians-Universität Münich, 81377 Münich, Germany; 3 Bernstein Center for Computational Neuroscience, Ludwig-Maximilians-Universität Münich, 81377 Münich, Germany; 4 Center for Vertigo and Balance Disorders, Ludwig-Maximilians-Universität Münich, 81377 Münich, Germany

**Keywords:** adaptive control, gain control, oculomotor, predictive control, sensorimotor transformation, smooth pursuit

## Abstract

The smooth pursuit eye movement system incorporates various control features enabling adaptation to specific tracking situations. In this work, we analyzed the interplay between two of these mechanisms: gain control and predictive pursuit. We tested human responses to high-frequency perturbations during step-ramp pursuit, as well as the pursuit of a periodically moving target. For the latter task, we found a nonlinear interaction between perturbation response and carrier acceleration. Responses to perturbations where the initial perturbation acceleration was contradirectional to carrier acceleration increased with carrier velocity, in a manner similar to that observed during step-ramp pursuit. In contrast, responses to perturbations with ipsidirectional initial perturbation and carrier acceleration were large for all carrier velocities. Modeling the pursuit system suggests that gain control and short-term prediction are separable elements. The observed effect may be explained by combining the standard gain control mechanism with a derivative-based short-term predictive mechanism. The nonlinear interaction between perturbation and carrier acceleration can be reproduced by assuming a signal saturation, which is acting on the derivative of the target velocity signal. Our results therefore argue for the existence of an internal estimate of target acceleration as a basis for a simple yet efficient short-term predictive mechanism.

## Significance Statement

Because of its modest complexity, analysis of the smooth pursuit control system offers a promising approach for understanding the principle mechanisms that transform visual perception into motor action. While previous studies investigated smooth pursuit gain control and predictive pursuit separately, here we investigated for the first time the interaction between them. We present strong evidence for the use of an internal estimate of target acceleration in the pursuit control circuit. Electrophysiological studies have shown that such a signal might be extracted from neuronal responses in the extrastriate visual cortex. Our results therefore suggest an extended functional role for this area in sensorimotor transformation. Further, we propose a physiologically plausible modification of the standard pursuit model, which reproduces the observed system behavior.

## Introduction

It has been known for a number of decades now that the smooth pursuit control system is not just a simple linear feedback mechanism for stabilizing the image of moving objects on the retina but incorporates a number of (often nonlinear) features to improve its performance in certain pursuit situations. One such feature is the so-called “gain control” mechanism, which adjusts the strength, or gain, of the visual–motor transmission, depending on the momentary tracking velocity (*V*; Keating and Pierre, 1996; Lisberger, 2010). Higher velocities yield higher gain, thereby adapting the system to better track typical movement changes of naturally moving objects (Ladda et al., 2007).

**Figure 1.**
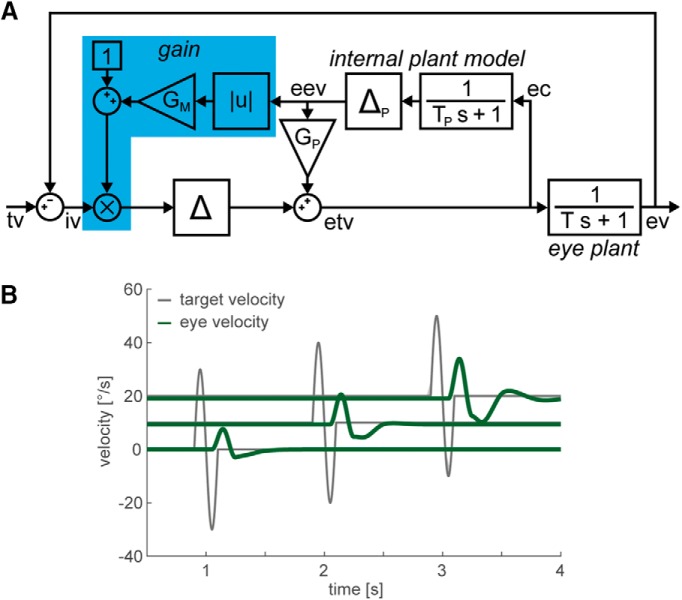
***A***, Smooth pursuit control circuit adapted from the study by Nuding et al. (2008). The model is based on the standard “internal feedback” model by Robinson et al. (1986), extended by a basic gain control mechanism (blue). Hereby, the retinal image velocity signal is modulated by an internal estimate of gaze velocity (Carey and Lisberger, 2004; Bayer et al., 2008; Lee et al., 2013). The retinal image velocity (iv) is the difference between target velocity (tv) and eye velocity (ev). The eye plant was modeled by a first-order low pass with time constant *T* = 200 ms. An efference copy (ec) of the motor command is projected to an internal model of the eye plant (with a time constant *T_P_* = *T*), yielding an estimate of the eye velocity signal (eev). This signal is used to transform iv into an estimate of target velocity (etv). For a steady-state pursuit gain of ∼0.9, *G_P_* was set 0.9. The gain control mechanism increases pursuit gain for higher velocities by multiplying iv with the factor $1+G_{M}\;\cdot \;\left|eev\right|$. The delay elements Δ and Δ*_P_* represent the processing latencies and were set to 150 ms. ***B***, Perturbation responses increase with eye velocity. Single-cycle high-frequency perturbations were imposed on target velocity (gray) during fixation, as well as pursuit with constant velocities of 10 and 20°/s. Green traces show the model responses with *G_M_* = 0.05 for the three different conditions.

Numerous studies analyzed the gain control mechanism by imposing high-frequency perturbations on the target movement (Schwartz and Lisberger, 1994; Churchland and Lisberger, 2002; Ono et al., 2010). Figure 1 illustrates the response to such perturbations for a standard model of the pursuit control circuit, including a nonlinear extension for gain control. Hereby, the retinal image velocity signal is modulated by an internal estimate of gaze velocity (Carey and Lisberger, 2004; Bayer et al., 2008; Lee et al., 2013). It has been shown that this relatively simple system configuration allows an adequate reproduction of experimentally observed perturbation responses (PRs) during the pursuit of targets moving with constant velocity (Nuding et al., 2008).

However, when the visual target is not moving with constant speed but instead exhibits a periodic velocity profile, another feature of the pursuit system comes into play. Within certain frequency and velocity ranges, the system is able to adapt almost perfectly to predictable target motion, performing zero-latency tracking and a steady-state pursuit gain close to unity (Bahill and McDonald, 1983a; Collewijn and Tamminga, 1984). Zero-latency tracking requires some predictive mechanism that compensates for the processing delays inherent to the pursuit control circuit. Deno et al. (1995) proposed the distinction between short- and long-term predictive mechanisms, depending on how much past target motion information is used. Whereas long-term mechanisms are assumed to be based on relatively complex cognitive mechanisms (Bahill and McDonald, 1983b; Barnes and Asselman, 1991; Shibata et al., 2005), short-term predictors are basically controllers with a phase-leading response. One possible implementation of such a short-term predictor, which was proposed by Yasui and Young (1984), introduces a derivative element into the pursuit control circuit to obtain a so-called “proportional-derivative” (PD)-controller.

In this work, we investigated the interplay between predictive pursuit and the gain control mechanism by analyzing responses to high-frequency perturbations during pursuit of a periodically moving target. Our finding of a nonlinear interaction between perturbation and carrier acceleration (*A*) argues for the existence of an internal estimate of target acceleration. We further show that a modified version of the PD-controller, combined with the standard gain control mechanism, reproduces the observed system behavior.

## Materials and Methods

### Subjects

Twelve healthy subjects participated [6 female, 6 male; mean (±SD) age, 30.2 ± 7.1 years] who had normal or corrected-to-normal vision and no relevant medical or psychiatric history. All subjects were naive regarding pursuit experiments. All subjects gave their informed consent before participating in the study, which conformed to the standards set by the Declaration of Helsinki and was approved by the Ethics Committee of the Faculty.

### Experimental setup

The experiments were performed in a completely dark room. Subjects sat in front of a 115 × 112 cm (width × height) back-projection screen at a viewing distance of 43 cm. The head was stabilized by a chin rest. Subjects were instructed to continuously follow the moving laser dot (size, 0.1°) with their eyes. The two-dimensional eye position was recorded using a custom-made monocular video-based eye tracker. An on-line pupil detection algorithm, running on a separate video computer, determined the coordinates of the pupil center in each of the images sampled at 100 Hz. The resolution of this system was <0.1°, and total accuracy was <0.5°. Eye movement data and the timing signals of the graphic computer were recorded on a central recording system (REX; Hays et al., 1982). Before each experiment a calibration was performed on the basis of 64 fixations to five target positions at the horizontal axis. The fixation positions expressed in image coordinates were detected and assigned to one of the target positions by using a cluster algorithm. The calibrated eye position was then computed as a linear function of the image coordinates.

### Paradigms

Each subject performed two different smooth pursuit tasks (in the respective order below). In both paradigms, the stimulus always moved horizontally in the frontoparallel plane.

#### Sinusoidal pursuit

**Figure 2.**
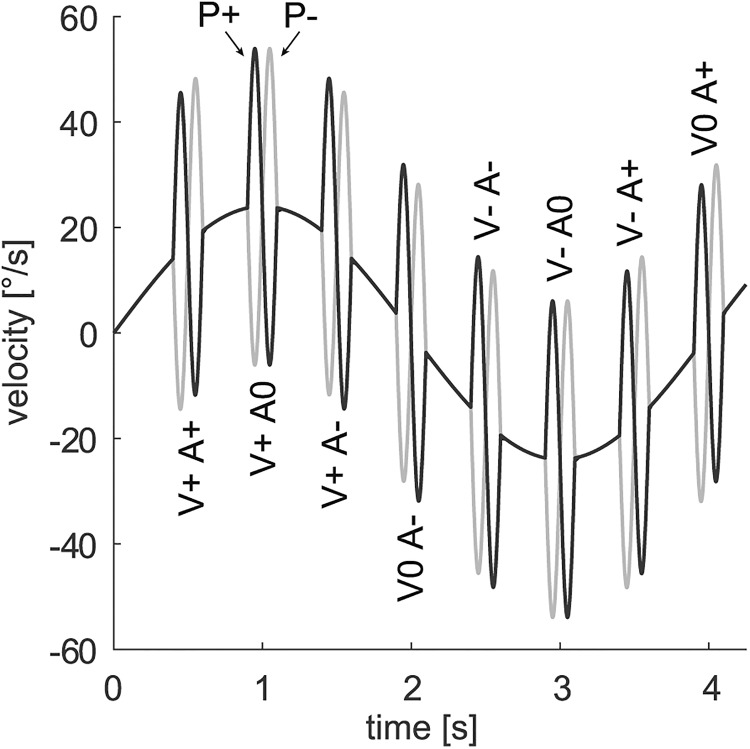
Possible forms of perturbation occurrence during sinusoidal pursuit. Initial perturbation acceleration was either to the right (*P+*) or to the left (*P−*). Perturbations occurred randomly at one of eight carrier phases. These were classified by the direction of carrier velocity *V* and the direction of carrier acceleration *A*, which was either rightward (*+*), leftward (*−*), or indefinable (0).

In this paradigm, the target was moving continuously with a sinusoidal velocity profile at a frequency of 0.25 Hz and maximal eccentricity of ±15° (peak velocity, 24°/s). Onto this “carrier” movement, high-frequency perturbations consisting of a single cycle of a 5 Hz sinus wave with ±30°/s peak velocity were randomly superimposed at the moment when carrier velocity was 0°/s, ±17°/s or ±24°/s. Perturbations were along the axis of target motion and occurred with the following two different types of phases: initial perturbation acceleration to the right direction (P+); or initial perturbation acceleration to the left direction (P−). Figure 2 illustrates the 16 different combinations of perturbation phase and occurrence. Movie 1 provides a short demonstration of this paradigm. The task consisted of 30 trials of 40 s length, interrupted by 1 s fixation periods at the center of the screen. As each cycle of the carrier contained one perturbation, a total of 300 perturbation responses per subject were recorded during this task.

Movie 1. Basic demonstration of the sinusoidal pursuit paradigm. The lower graph shows an example target velocity trace where high-frequency perturbations (*P+* and *P−*) were imposed at four different carrier phases on the sinusoidal carrier. The green dot marks the current velocity of the red target spot in the top graph. 10.1523/ENEURO.0343-16.2017.video.1

#### Step-ramp

In this paradigm, we analyzed responses to the same perturbations described above during the pursuit of a target moving with constant velocity of ±17 or ±24°/s (Movie 2). After reaching the maximal eccentricity of 30°, the target made a step back to the center of the screen. In a fifth type of trial, the so called “0°/s step-ramp,” the subjects performed fixation, as the target stayed at the center of the screen. The trials had a length of 3 s and appeared in randomized order. To prevent catch-up saccades after target onset, the target first made a step in the opposite direction. The step was arranged so that the target crossed the initial fixation position after 200 ms. For avoiding predictive behavior, perturbations occurred either 400 or 800 ms after target onset. Furthermore, trials without perturbation were randomly included. Also in this task, a total of 300 perturbation responses per subject were recorded.

Movie 2. Basic demonstration of the step-ramp paradigm. The bottom graph shows a target velocity trace for rightward pursuit with an imposed *P+* perturbation. The green dot marks the current velocity of the red target spot in the top graph. 10.1523/ENEURO.0343-16.2017.video.2

### Data analysis

Eye velocity was computed by linearly interpolating the 100 Hz eye position traces to a sampling rate of 1 kHz, filtering with a symmetrical Gaussian low-pass filter with a cutoff frequency of 10 Hz and subsequent 2-point differentiation. Eye velocity phases exceeding target carrier velocity by >50°/s were classified as saccades and were cut out from the data including a margin of 40 ms before and after the saccade. The resulting gaps in the velocity traces were not interpolated to avoid a potential distortion of the perturbation responses.

For the step-ramp conditions, trials were extracted from the data and sorted according to their carrier velocity and perturbation phase. Eye velocity traces of trials for the two different time points of perturbation occurrence were pooled together. The steady-state gain was determined by dividing the average eye velocity during the time interval of 300–800 ms after target onset by target carrier velocity. The onset latency *t_on_* was determined for the two pursuit velocities by least-squares fitting of the function $f_{on}=K(1-e^{-\frac{t-t_{on}}{T}})$ to the averaged eye velocity traces. Steady-state gain and onset latency were averaged for the two different pursuit velocities.

For the sinusoidal conditions, trials were extracted from the data and sorted according to the 16 different types of perturbation phase and occurrence (Fig. 2). For further analysis, a sine wave was least-squares fitted in amplitude and phase to the averaged eye velocity traces. The steady-state gain was computed by dividing the amplitude of the fitted sine wave by 24°/s. It was averaged across the different conditions. The phase delay was defined by the peak of the cross-correlation function of the target and eye velocity traces.

**Figure 3.**
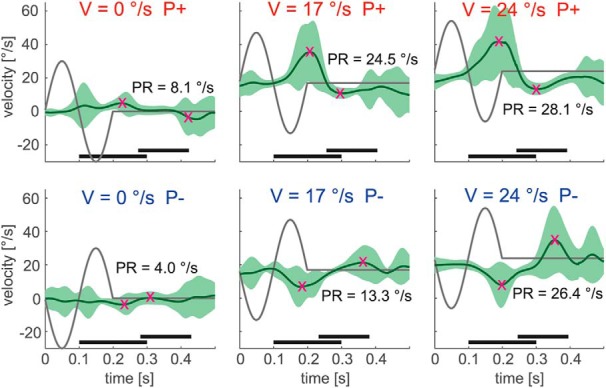
Responses to high-frequency perturbations in an example subject during fixation (*V* = 0°/s) and step-ramp smooth pursuit (*V* = 17 and 24°/s). Initial perturbation acceleration was either to the right (*P+*) or to the left (*P−*). Green traces show the average eye velocity, with the SD marked light green. Dark gray traces show target velocity. Magenta crosses mark peaks and troughs of the PR. For *P+* perturbations, the maximum eye velocity was detected in the time interval of 100–300 ms (bottom black bar) after perturbation onset (*t* = 0 s). The minimum was detected in the time interval of 50–200 ms (top black bar) after the maximum. In the *P−* case, time intervals for maximum and minimum were transposed accordingly.

Analogous to previous work (Schwartz and Lisberger, 1994; Churchland and Lisberger, 2002; Ono et al., 2010), the influence of perturbations on the eye movements was quantified using a “peak-to-trough” approach. Specifically, we defined the “perturbation response” as the difference between the maximum and the minimum eye velocity after occurrence of a perturbation. For P+ perturbations, the maximum eye velocity was detected in the time interval of 100–300 ms after perturbation onset. The minimum was detected in the time interval of 50–200 ms after the maximum. The length of these time intervals was chosen to cover the actual range of response latencies (Fig. 3). In the P− case, time intervals for maximum and minimum were transposed accordingly. In the sinusoidal conditions, the fitted sine waves were subtracted from the eye velocity traces for PR determination.

Statistical analysis was performed using SPSS version 23 (IBM; RRID: SCR_002865). Differences between PRs were analyzed using repeated-measures ANOVA and linear regression analysis (with “±” denoting the 95% confidence interval). The sphericity assumption was tested using Mauchly’s test. In the case of violation, the multivariate approach was used. Effects with α levels <0.01 were considered to be significant.

All simulations were conducted with Simulink R2015b (MathWorks; RRID: SCR_001622) using the Dormand–Prince variable step solver (ode45) with default settings. The input signals and the algorithms for data analysis corresponded to the experiment. Model parameters were fitted to minimize the sum of squared errors using the Nelder–Mead simplex algorithm for nonlinear optimization (Nelder and Mead, 1965). To estimate the variance of the model parameters, the Jackknife resampling technique (Efron and Stein, 1981) was used. Hereby, the optimization routine was repeated *n* times while leaving out one of the *n* observations for each iteration. The variance of the estimator is then estimated as follows:

$$\sigma^{2}=\frac{n-1}{n}\overset{n}{\underset{i=1}{\sum }}{\left({\overset{¯}{x}}_{i}-{\overset{¯}{x}}_{\left(\cdot \right)}\right)}^{2}$$

where ${\bar{x}}_{i}$ is the estimator based on leaving out the *i*th observation, and ${\bar{x}}_{\left(\cdot \right)}$ is the estimator based on all observations.

## Results

To analyze the interaction of a smooth pursuit predictive mechanism with gain control, we investigated the influence of high-frequency target perturbations on eye movements for the following two fundamentally different paradigms: aperiodic step-ramp and periodic, predictable sinusoidal pursuit. In general, all subjects performed well during both tasks. For step-ramp, the mean (±SD) steady-state gain was 0.88 ± 0.07, and the mean onset latency was 141.3 ± 15.1 ms. During sinusoidal pursuit, zero-latency tracking with an average steady-state gain of 1.03 ± 0.09 could be observed in all subjects. The general tracking behavior in both paradigms was therefore in accordance with that in previous reports (Barnes, 2008).

### Responses to high-frequency perturbations during step-ramp and sinusoidal pursuit

**Figure 4.**
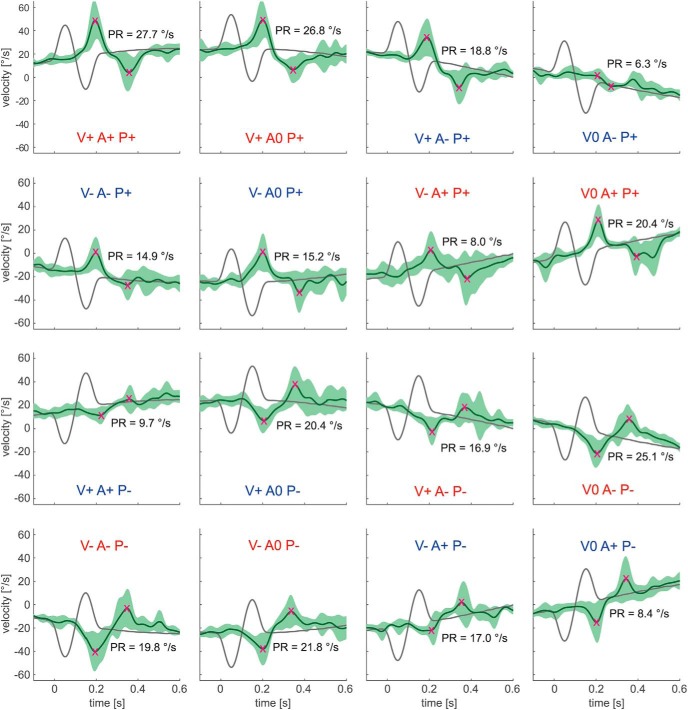
Responses to high-frequency perturbations in an example subject during sinusoidal pursuit. All 16 conditions, as described in Figure 2, are illustrated. Green traces show the averaged eye velocity, with the SD marked light. Dark gray traces show target velocity. Magenta crosses mark peaks and troughs of the PR. For PR determination the carrier was subtracted. The labels for ipsidirectional perturbations are colored red, the labels for contradirectional perturbations are colored blue.

Figures 3 and 4 show the averaged eye velocity traces during perturbation occurrence for an example subject. In the step-ramp paradigm, for both kinds of perturbation phases a strong increase of the PR with target carrier velocity could be observed. During sinusoidal pursuit, by contrast, it was difficult to recognize a clear dependence of the PR on perturbation phase or occurrence on a single-subject level.

**Figure 5.**
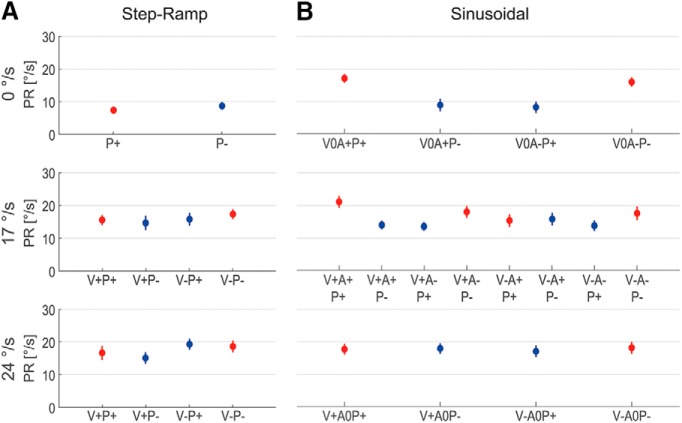
***A***, ***B***, PR during step-ramp (***A***) and sinusoidal pursuit (***B***). Conditions are labeled according to the initial perturbation direction (*P+*, rightward; *P−*, leftward) and the directions of carrier velocity (*V+*, rightward; *V−*, leftward) and acceleration (*A+*, rightward; *A−*, leftward). Sinusoidal 0 and 24°/s conditions correspond to the turning points and the center points of the carrier movement, respectively. Peak-first (***A***) and ipsidirectional (***B***) perturbations are colored red, and peak-last and contradirectional perturbations are colored blue. Vertical lines mark SEs. Note the difference between ipsidirectional and contradirectional PRs in the sinusoidal 0 and 17°/s conditions.

The mean PRs for all tested conditions averaged over the 12 subjects are shown in Figure 5. As both paradigms are fully symmetric regarding leftward and rightward target movement, each tested condition has a counterpart in the other direction. To test whether perturbation responses superimpose linearly with the steady-state response or whether they depend in a nonlinear way on the direction of the carrier velocity, we adopted for the step-ramp task the previous definition of Churchland and Lisberger (2002) and refer to “peak-first” perturbations if the target first accelerates and then decelerates. Otherwise, the perturbation phase is called “peak-last.” For the fixation trials (0°/s), both kinds of perturbation lead to initial acceleration of the target. Consistent with previous work (Churchland and Lisberger, 2002), in this condition we refer to peak-first perturbations if the target first accelerates in the positive direction (right), and to peak-last perturbations if the target first accelerates in the negative direction (left).

In sinusoidal pursuit, not only the velocity but also the acceleration of target carrier movement varied between conditions (Fig. 2). As will be apparent later, it is useful to distinguish whether the acceleration of the perturbation complies with carrier acceleration or not. We therefore classified the perturbation phase as “ipsidirectional” (ipsi-dir) if the initial acceleration of the perturbation has the same direction as the carrier acceleration. Otherwise the perturbation phase is called “contradirectional” (contra-dir). For the sinusoidal trials at 24°/s, where acceleration is zero, we refer to ipsi-dir if the initial perturbation of the perturbation has the same direction as carrier acceleration immediately before perturbation occurrence. Otherwise, the perturbation phase is called contra-dir. This classification of sinusoidal 24°/s trials, as well as the peak-first/peak-last classification of fixation trials, is to some extent arbitrary. However, we chose to use it for simplification purposes and for comparability with previous works.

We next analyzed, separately for each carrier velocity, the dependence of PRs on carrier movement direction and perturbation phase. In the step-ramp task (Fig. 5*A*), the ANOVA with factors carrier velocity direction and perturbation phase (peak-first/peak-last) showed no significant differences between leftward and rightward movement (17°/s: *F*_(1,11)_ = 1.96, *P* = 0.19; 24°/s: *F*_(1,11)_ = 1.79, *P* = 0.21) and between peak-first and peak-last perturbations (0°/s: *F*_(1,11)_ = 1.63, *p* = 0.23; 17°/s: *F*_(1,11)_ = 0.09, *p* = 0.78; 24°/s: *F*_(1,11)_ = 0.10, *p* = 0.76).

For the sinusoidal paradigm (Fig. 5*B*), the different PRs were analyzed with respect to the three factors perturbation phase, carrier velocity direction, and carrier acceleration direction. For 0 and 17°/s, ANOVA revealed significant difference in PRs for the two kinds of perturbation phases, ipsi-dir and contra-dir (0°/s: *F*_(1,11)_ = 22.94, *p* < 0.01; 17°/s: *F*_(1,11)_ = 50.14, *p* < 0.001). For 24°/s, there was no significant difference between the two kinds of perturbation phases (*F*_(1,11)_ = 0.51, *p* = 0.49). For all carrier velocities, no other main effects as well as no significant interactions between the factors were found. In summary, for both paradigms perturbation responses did not differ between leftward and rightward carrier movement. The main effects of the perturbation phase occurred in the sinusoidal case but not in step-ramp.

For further analysis, we pooled across the levels of the nonsignificant factors carrier velocity direction and carrier acceleration direction and will consider from now on only differences in target carrier velocity and perturbation phase. This reduction of the number of factors was only possible because we defined our perturbation phases for sinusoidal pursuit in a way that differed from the definitions of peak-first/peak-last perturbation phases in the step-ramp experiments. For example, both the rightward perturbation superimposed on the rightward and increasing carrier velocity (Fig. 5*B*; 17°/s condition V+A+P+) and the rightward perturbation superimposed on the rightward, but decreasing, carrier velocity (condition V+A−P+) would have been classified as peak-first according to the existing definition, since in both cases the initial perturbation had the same direction as the actual carrier velocity. However, the two responses differed clearly from each other. Thus, using the step-ramp definition of perturbation phases as a factor in the ANOVA would not have allowed eliminating the factor direction of carrier acceleration.

**Figure 6.**
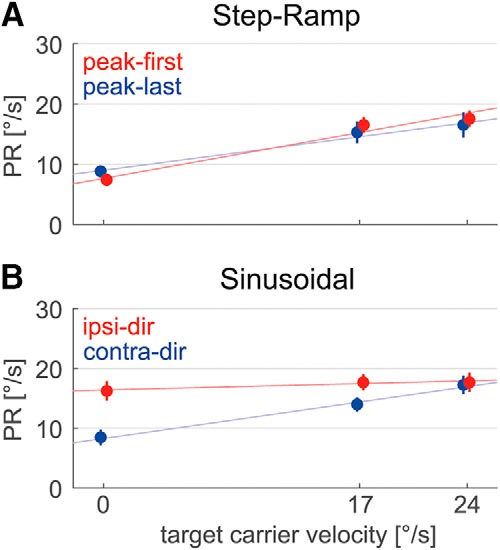
Dependence of mean PRs on the three different carrier velocities and the perturbation phase. ***A***, ***B***, In step-ramp (***A***), perturbations were either peak-first (red) or peak-last (blue). During sinusoidal pursuit (***B***), perturbations were classified as ipsidirectional (red) or as contradirectional (blue). Thin colored lines illustrate the results from linear regression analysis. Vertical lines mark SEs.

Figure 6 shows the dependence of perturbation responses on carrier velocity and perturbation phase for the two different paradigms. In the step-ramp case, for both kinds of perturbation phases PRs increased linearly with carrier velocity (linear regression analysis: peak-first: *β* = 0.45 ± 0.15, *p* < 0.001; peak-last: *β* = 0.32 ± 0.19, *p* < 0.01). During sinusoidal pursuit, for contra-dir perturbations PRs exhibited a linear increase with carrier velocity (*β* = 0.36 ± 0.16, *p* < 0.001). The ipsi-dir perturbations, on the contrary, showed no dependence on carrier velocity (*β* = 0.06 ± 0.18, *p* = 0.45). ANOVA for the step-ramp peak-last and sinusoidal contra-dir conditions with factors paradigm and carrier velocity showed no significant difference between the two paradigms (*F*_(1,11)_ = 0.21, *p* = 0.65).

The observation that responses to perturbations in target velocity superimposed on sinusoidal carrier velocities depend systematically on the perturbation phase clearly points toward a nonlinearity of the system. Moreover, the results of our analysis suggest that the most important factor accounting for the difference between the two kinds of perturbation phases is a relation between the initial direction of the perturbation and the actual or recent carrier acceleration. Therefore, we tried to explain the observed behavior by a nonlinearity acting on an internal acceleration signal. The assumption of such an internal acceleration signal occurring in a pursuit control circuit seems to be natural, because (1) a PD-controller would require such a signal, and (2) because a PD-controller is a straightforward mechanism to minimize phase delays, which are introduced by neuronal processing and the low-pass characteristic of the plant. We will next present a modified version of the PD-controller, which, combined with the standard gain controller, allows a reproduction of our findings.

### A bounded PD-controller model of smooth pursuit

**Figure 7.**
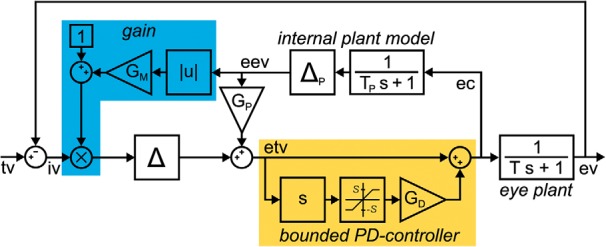
A bounded PD-controller model of smooth pursuit. This control circuit is an extension of the basic gain control model from Figure 1. The block labeled “*s*” represents the derivative element of the bounded PD-controller. The derivative of the estimated target velocity signal etv is bounded by a saturating element (with thresholds *−S* and *S*) and amplified by *G_D_*. By summation with etv the motor command is generated.

Figure 7 illustrates the gain control pursuit model from Figure 1 extended by a bounded PD-controller for predictive pursuit. The delay of the lag element, which represents the combined neuronal und muscular processing latencies, was set to Δ = 140 ms to replicate the observed onset latency. For stability reasons, the lag of the internal plant model Δ*_P_* was set to equal Δ (Robinson et al., 1986). The gain of the internal plant model (*G_P_*) was set to *G_P_* = 0.9 to replicate the observed steady-state gain in the step-ramp paradigm.

**Figure 8.**
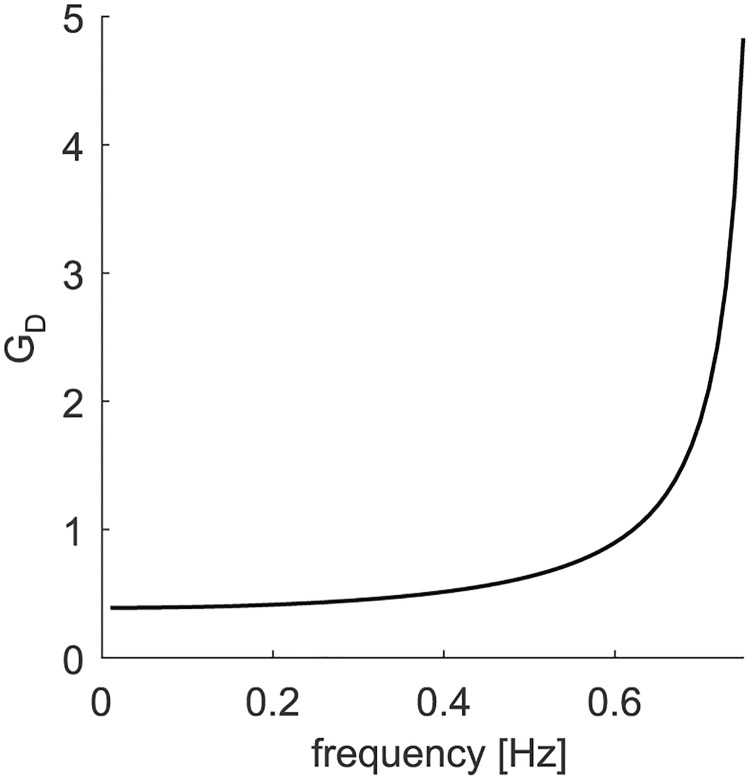
The gain of the derivative signal *G_D_* as a function of target carrier frequency.

The PD-controller uses the derivative of the delayed target velocity signal to compensate for the processing delay Δ. Strictly speaking, this applies only to periodic signals with dominant frequency *f*, as the differentiation corresponds to a phase lead of $\frac{\pi }{2}$ (Kuo and Golnaraghi, 2002). For certain ranges of *f* and Δ, the nondelayed target velocity signal can be reconstructed by adding the appropriately weighted delayed derivative of this signal. In the following paragraph, we will derive the value of the weight of the derivative gain (*G_D_*), which is required to perform zero-latency tracking during sinusoidal pursuit. We first consider the unbounded case (*S* = ∞). *G_D_* determines the gain of the derivative signal and thereby the magnitude of the phase lead imposed on the system. To replicate a fully compensating predictive pursuit behavior, the phase lead of the PD controller needs to match the sum of the phase delays produced by the lag element and the eye plant for a given frequency of the target signal. The PD-controller has the transfer function $F_{PD}=1+G_{D}s$ and the phase response $\varphi_{PD}=arctan({\omega G}_{D})$, with ω=2πf. The eye plant is modeled by a first-order low-pass filter with transfer function $F_{eye}=\frac{1}{Ts+1}$ and phase response $\varphi_{eye}=-arctan(\omega T)$. The lag element produces a constant signal delay Δ, which translates to the phase delay $\varphi_{\Delta }=-\omega \Delta$. For full phase compensation, it holds that $\varphi_{PD}+\varphi_{eye}+\varphi_{\Delta }=0$. Thus, the ideal dependence of *G_D_* on frequency would be expressed by $G_{D}=\frac{1}{\omega }tan(\arctan\left(\omega T\right)+\omega \Delta )$. Figure 8 shows this ideal *G_D_* as a function of carrier frequency *f* for our parametrization. *G_D_* exhibits modest increase up to a frequency of ∼ 0.6 Hz and then rises asymptotically toward the pole at 0.75 Hz (for how the choice of *G_D_* might be implemented, see also Discussion).

To ensure adequate phase compensation by the bounded PD-controller during predictive pursuit, a minimum for the saturating threshold for the derivative signal $S=\pm S_{min}\,\;\cdot \;G_{S},G_{S}\geq 1$ was introduced. *S*_min_ should equal the maximum of the derivative of the estimated target velocity *etv* (i.e., the peak estimated target acceleration) during zero-latency pursuit of the sinusoidal target signal with frequency *ω* and amplitude *A*. Under the assumption of perfect tracking, image velocity becomes zero and ${etv}_{iv=0}=G_{P}\,\;\cdot \;A\,\;\cdot \;\omega \,\;\cdot \;\sin\left(\omega t\right)$. With this, we get $S_{min}=G_{P}\,\;\cdot \;A\,\;\cdot \;\omega 2$.

**Figure 9.**
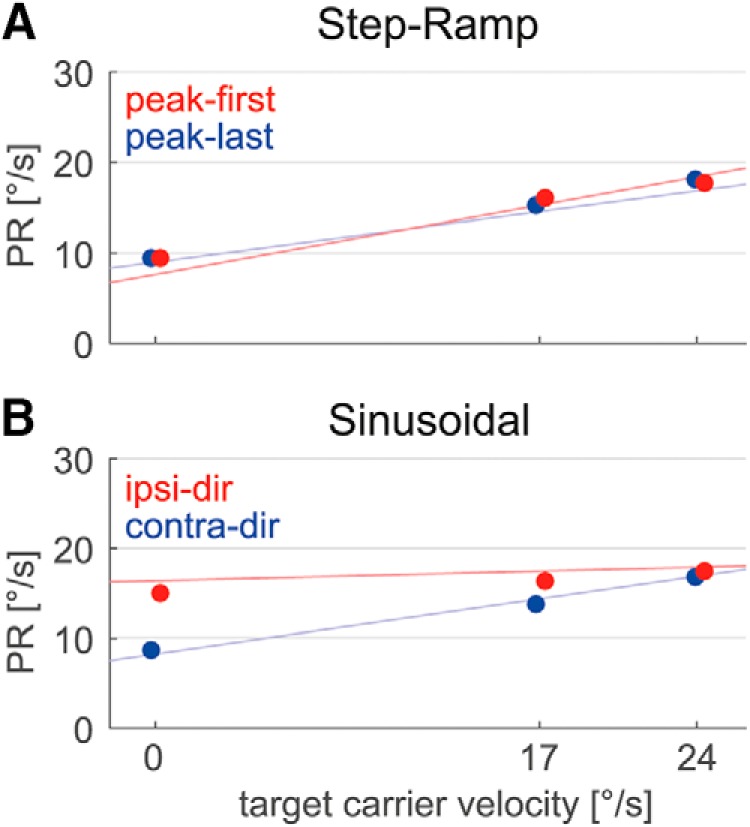
PRs for the bounded PD-controller model. ***A***, In step-ramp, perturbations were either peak-first (red) or peak-last (blue). ***B***, During sinusoidal pursuit, perturbations were classified as ipsidirectional (red) or contradirectional (blue). Regression lines from Figure 6 are shown for comparison.

Least-squares fitting the parameters for an optimal approximation of the PRs of the model to the combined data from both the sinusoidal and the step-ramp condition yielded mean squared errors of 6.05 (°/s)^2^ in the sinusoidal condition and 6.87 (°/s)^2^ in the step-ramp condition for *T* = 279 ms, *G_M_* = 0.040, and *G_S_* = 1.46. Figure 9 shows that the resulting smooth pursuit model, which combines the standard gain control mechanism with a bounded PD-controller for predictive pursuit, replicates the observation that PRs increase with increasing carrier velocity for ipsidirectional but not for contradirectional perturbations.

For further validation, we next fitted the parameters of the model separately to each of the two conditions. We used the Jackknife resampling technique to estimate the variance of the model parameters. Table 1 shows that the parameters obtained from the combined fit (first column) lie in the 95% confidence intervals of both the fit to the sinusoidal (second column) and the fit to the step-ramp data (third column). Hence, the estimated parameters did not strongly differ between the two subsets of data.

**Table 1.** Parameter values of the bounded PD-controller model after fitting the data from both the sinusoidal and the step-ramp conditions, the sinusoidal condition only, and the step-ramp condition only

	SIN + STEP	SIN only	STEP only
Δ, Δ*_P_*	140 ms		
*T*, *T_P_*	279 ms	245 (±59) ms	285 (±51) ms
*G_P_*	0.9		
*G_M_*	0.040	0.029 (±0.018)	0.042 (±0.014)
*G_D_*	0.467	0.424	0.475
*G_S_*	1.46	1.62 (±0.88)	1.19 (±0.49)

Note that Δ, Δ*_P_*, and *G_P_* were not fitted, as well as *G_D_* (see Results). The values in parentheses denote the 95% confidence interval. SIN, Sinusoidal; STEP, step-ramp.

**Figure 10.**
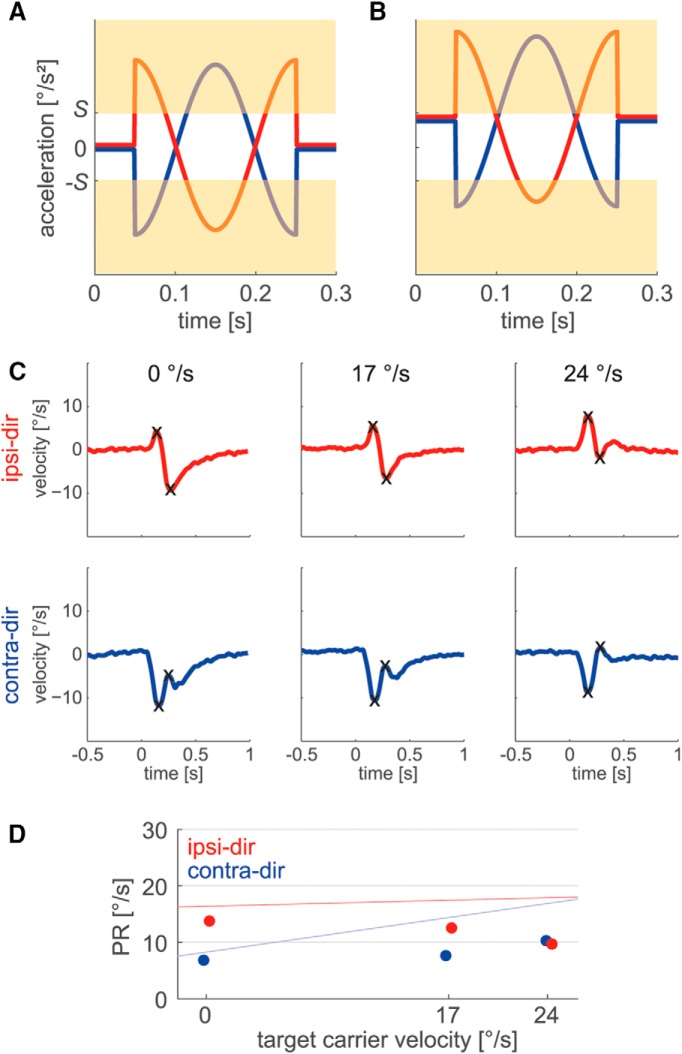
***A***, ***B***, Derivative signal during perturbation occurrence. Signal components exceeding thresholds *S* or *−S* are bounded by the saturating element (as indicated by the beige area). ***A***, ***B***, For a carrier acceleration of 0°/s^2^ (***A***), ipsi-dir (red) and contra-dir (blue) perturbations are affected similarly. For maximal carrier acceleration (***B***), ipsi-dir and contra-dir perturbations are affected asymmetrically by the signal saturation. ***C***, Responses to ipsi-dir (red) and contra-dir (blue) perturbations of the bounded PD-controller model with *G_M_* = 0 for three different carrier velocities during sinusoidal pursuit. For better illustration, the sinusoidal carrier has been subtracted. At 17°/s condition *V+A+* is shown (Fig. 2). Black crosses mark peaks and troughs of the PR. ***D***, PRs for the bounded PD-controller model with *G_M_* = 0 during sinusoidal pursuit. Regression lines from Figure 6 are shown for comparison.

Our simulations showed that the divergent responses to perturbations during sinusoidal pursuit can be explained by the proposed nonlinear bounded PD-controller. But how does the saturation of the derivative signal lead to the observed distortions depending on carrier velocity and perturbation phase? For a better understanding of this mechanism, we further analyzed the impact of the saturation on the two different perturbation phases. For a carrier acceleration of 0°/s^2^ (which corresponds to maximal carrier velocity), both lower and upper bounds have a symmetrical effect on the two types of perturbation phases (Fig. 10*A*). With an increase of carrier acceleration, however, the positive fractions of acceleration caused by the two types of perturbations are more and more truncated, whereas the negative fractions become larger (Fig. 10*B*). Figure 10, *C* and *D*, illustrate the consequences of this asymmetrical distortion on the PRs of the bounded PD-controller model with a deactivated gain modulation &(*G_M_*) mechanism (*G_M_* = 0). For a carrier velocity of 24°/s, which corresponds to zero carrier acceleration (Fig. 10*A*), ipsi-dir and contra-dir perturbations induce similar effects on eye velocity, resulting in almost equal PR values for this condition. However, for lower carrier velocities, corresponding to nonzero carrier acceleration (Fig. 10*B*), the asymmetrical cutoff of the internal acceleration signal leads to differing responses for the two types of perturbation phases. Due to the truncation of positive signal fractions and the increase of negative fractions, the saturation causes a reduction of the accelerating component and an enhancement of the decelerating component of the perturbation response (Fig. 10*C*). For ipsi-dir perturbations, this results in a slight decrease of the initial peak component and substantial enlargement of the secondary trough, leading to an overall increase of the PR value with decreasing carrier velocity. In the case of contra-dir perturbations, on the other hand, the initial trough is amplified and the secondary peak becomes a bit smaller, leading to an overall decrease of the PR value. For maximal (as well as minimal) carrier acceleration, corresponding to a carrier velocity of 0°/s, the asymmetry is largest. Inclusion of the gain modulation mechanism into the system leads to an equal increase of PR with higher carrier velocity for both kinds of perturbation phases (Fig. 9*B*). The apparent independence of ipsi-dir PRs on carrier velocity in the sinusoidal condition therefore rather results from the superposition of two separate mechanisms with similar effects but opposite directions of action: gain modulation leads to a linear increase of PR for higher carrier velocities, while the saturation in the PD-controller results in a comparable increase of PR with lower carrier velocities.

## Discussion

High-frequency perturbations imposed on periodic sinusoidal target movement revealed an eminent nonlinearity in the predictive mechanism of the smooth pursuit control system. Whereas for contra-dir perturbations responses showed a similar dependence on target carrier velocity as observed during the step-ramp condition, ipsi-dir perturbation responses were large for all target velocities. This effect may be explained by combining two independent nonlinear system features: gain control and a bounded PD-controller. The resulting smooth pursuit system model can be tuned to perform realistic zero-latency tracking of periodic input signals and reproduces the observed perturbation responses during step-ramp as well as sinusoidal pursuit. The predictive mechanism is based on an internal estimate of target acceleration. Electrophysiological studies have shown that such a signal might be extracted from neuronal responses in the medial temporal cortical area (Lisberger and Movshon, 1999; Price et al., 2005; Schlack et al., 2007, 2008). Apart from providing an image velocity signal for tracking eye movements, the extrastriate visual cortex might therefore also play a role in adaptive control features of pursuit.

### Smooth pursuit gain control

In comparison with previous studies, we used a large perturbation amplitude of ±30°/s in our experiment. The effect size reported here is therefore not directly comparable to prior work. Nevertheless, previous studies have also found a linear increase in perturbation responses with step-ramp target velocity. Keating and Pierre (1996) found in monkeys a linear increase of gain to multicycle perturbations (4 Hz, ±8°/s) for target velocities between 1 and 15°/s. This finding was later confirmed for human subjects using a similar multicycle perturbation paradigm (Nuding et al., 2008). In another human study, Churchland and Lisberger (2002) reported for single-cycle peak-first perturbations (5 Hz, ±5°/s) and target velocities of 0, 5, and 10°/s a linear increase with slope β = 0.24 (adjusted to our PR measure). The larger β value that we observed is most likely related to the greater perturbation amplitude. Interestingly, for peak-last perturbations (2.8 Hz, ±19°/s) Churchland and Lisberger (2002) found no significant difference in perturbation responses during fixation and 10°/s target velocity pursuit. This observation is in contrast with our finding of similar PR increases with step-ramp target velocity. One possible explanation for this difference is that our perturbations started later (400–800 ms) than the perturbations applied by Churchland and Lisberger (2002), which occurred 250 ms after the onset of target motion. A potential aftereffect of the impulse-like target acceleration at target movement onset on an internal acceleration signal would have decayed more at the later perturbation times in our experiment. Thus, the nonlinear saturation effects induced by our hypothesized bounded PD-controller are expected to decay with later occurring perturbations. This might explain why we did not observe differences between peak-first and peak-last perturbations in our step-ramp paradigm. However, in a monkey study with perturbation occurrence between 100 and 600 ms after target onset, Schwartz and Lisberger (1994) found an increase in perturbation responses with target velocity for both peak-first, and peak-last perturbations, which is in line with our results.

### Predictive mechanisms in pursuit control

A number of findings argue for the existence of one or more long-term predictors in the pursuit system. For instance, after the disappearance of a sinusoidally moving target (Whittaker and Eaholtz, 1982) or an unexpected change in its motion (van den Berg, 1988), a continuation of some oscillatory eye movements at the frequency of the preceding target movement can be observed. Also, during pursuit of triangular target motion, eye velocity maintains its previous movement pattern for >500 ms after an unexpected modification of the target trajectory (Deno et al., 1995). Long-term predictors are related to anticipatory eye movements and, therefore, are probably closely linked to higher cognitive functions (Kowler, 1989; Barnes et al., 2000; Barnes, 2008). Accordingly, it is assumed that relatively complex mechanisms, including periodicity estimators and memory elements, underlie this kind of predictive system feature (Bahill and McDonald, 1983b; Barnes and Asselman, 1991; Shibata et al., 2005).

A short-term predictor, on the other hand, uses only local information in the form of target position and its derivatives to extrapolate into the future. One particular characteristic of smooth pursuit is usually considered as an argument for the existence of a short-term predictive element in pursuit control: during the tracking of a nonpredictable target signal (e.g., the sum of nonharmonic sinusoids), a large phase lead for the low-frequency components can be observed (Yasui and Young, 1984; van den Berg, 1988; Deno et al., 1995). Yasui and Young (1984) showed that this finding may be reproduced by introducing a PD-controller into the pursuit circuit.

For a purely sinusoidal target carrier movement, the distinction between short- and long-term predictors is not trivial as both kinds of mechanisms are potentially capable of performing zero-latency tracking. There is evidence for the existence of both features in predictive pursuit control. We hypothesize therefore that short- and long-term predictive mechanisms might interact in a hierarchical cascade of control features, where the PD-controller constitutes the lowest level. With an increasing demand of cognitive processes to predict the future course of target movement, additional higher-level long-term predictors set in.

The important new aspect of the present study is the analysis of the nonlinear effects that result from a saturation of the derivative component of the PD-controller. Previous models such as the one of Yasui and Young (1984) did not include such a component and led to infinite signal values for steps in target velocity and potential instability due to an accentuation of high-frequency noise. A different form of short-term predictor, which is known as the “phase-lead controller” in the control theoretical literature, was proposed by van den Berg (1988). It used an integrator instead of a derivative element, thereby avoiding the stability problems of a PD-controller. However, this type of controller is also fully linear and can therefore not reproduce the nonlinear effects of perturbation phases on PRs during sinusoidal pursuit. Introducing a saturation element into the PD-controller, on the other hand, counteracts its stability problems and results in a physiologically plausible predictive mechanism that is able to reproduce the observed perturbation responses.

### Further evidence for a derivative control element

Various behavioral experiments have previously presented evidence for a derivative control element in the smooth pursuit system. In a step-ramp paradigm where the initial stimulus onset was either an abrupt change in velocity or a gradual acceleration, Krauzlis and Lisberger (1994) found differences in initial eye movement onset, which indicated an influence of target acceleration on the system. Other studies confirmed this acceleration sensitivity, albeit observing a stronger effect during steady-state pursuit compared with the onset (Watamaniuk and Heinen, 2003; Bennett et al., 2007).

Further evidence for a derivative element in the pursuit system might be provided by the finding of an upper frequency limit for zero-latency tracking. During pursuit of sinusoidal motion with very small amplitudes, Martins et al. (1985) observed a breakdown of effectiveness for frequencies >0.5 Hz, although the velocity and acceleration of target motion were low. Baloh et al. (1988) found an increase of about fourfold to fivefold in phase delay during sinusoidal pursuit at 0.8 Hz compared with pursuit at 0.4 Hz, even if stimulus amplitude was decreased a factor of four. Also Waterston et al. (1992) reported a significant increase of phase delay for frequencies >0.4 Hz, which persisted remarkably independent of the sinusoidal target amplitude. Figure 8 shows that, given the assumed signal delays and plant characteristics, exact phase compensation by means of a PD-controller would work up to an upper frequency limit of ∼0.75 Hz. However, this limit is only approached for infinite gains of the derivative signal (*G_D_*), which is evidently impossible to achieve. The finding that the actual breakdown of effective phase compensation occurs already at 0.4 Hz might have a different explanation: the gain of the actual implementation of a linear PD-controller may increase less with frequency than is required for accurate phase compensation. Since the ideally required derivative gain depends only a little on frequency for *f* < 0.4 Hz (Fig. 8), we propose a simple approximation by assuming a constant *G_D_* in combination with a carrier acceleration-dependent saturation. Because we did not test multiple carrier frequencies, this feature of the model must be considered as a so far unconfirmed hypothesis.

Subsequent studies will be necessary to further test our model. For example, it might be interesting to test the perturbation responses for a condition with constant target acceleration. The bounded PD-controller model predicts in this case similar differences in PRs between the ipsi-dir and contra-dir conditions for all carrier velocities, as we have observed for a carrier velocity of 0°/s.

## Synthesis

Reviewing Editor: Bradley Postle, University of Wisconsin Decisions are customarily a result of the Reviewing Editor and the peer reviewers coming together and discussing their recommendations until a consensus is reached. When revisions are invited, a fact-based synthesis statement explaining their decision and outlining what is needed to prepare a revision will be listed below. The following reviewer(s) agreed to reveal their identity: Xin Huang, Antonino Casile.

The two reviewers are generally happy with the revision and accompanying letter, and have only relatively minor points. It is hoped that a second revision and accompanying response letter will be sufficiently clear that it won't need to be sent out for another round of peer review.

R1: Two remaining points.

1. One issue regards the cross-validation. The authors did this by comparing the model parameters fitted based on the full data set, data from the sinusoidal condition and data from the step-ramp condition. By showing that the model parameters fitted based on the full data set are within 95% confidence interval of the parameters based on the subset of the data, the authors concluded that the estimated parameters did not strongly differ between the two subsets of data. Although it is helpful to see the model parameters fitted using the full and the subsets of the data, I think these results do not directly address the issue of cross-validation. The question is how well the model can fit novel data.

It is also stated on page 12, the third paragraph that “Least-squares fitting the parameters for an optimal approximation of the model's PRs to the combined data from both, the sinusoidal, and the step-ramp conditions yielded mean squared errors of 6.05 in the sinusoidal condition and 6.87 in the step-ramp condition”. The testing data used to obtain the MSE described on page 12 were also used to fit the model parameters. This does not seem to be a conventional approach to do cross-validation, which would typically take a subset of the data (e.g. 90% of the trials) to do the model fitting, and then test the model with the data that have not been used to obtain the model parameters.

2. The authors described their model prediction for constant acceleration condition. I agree with the authors that testing this prediction would require future research and does not need to be included in this manuscript. But I think it would be meaningful to describe their specific model prediction in the Discussion of this paper. The prediction has already been described in the rebuttal letter, so this does not require a significant amount of work.

R2: The only very minor concern that I have is that I would like the Authors to better describe their resampling procedure in the Methods section (e.g. how many observations did they leave out at each “cycle” of the jackknife algorithm?).
